# Benchmarking workflows to assess performance and suitability of germline variant calling pipelines in clinical diagnostic assays

**DOI:** 10.1186/s12859-020-03934-3

**Published:** 2021-02-24

**Authors:** Vandhana Krishnan, Sowmithri Utiramerur, Zena Ng, Somalee Datta, Michael P. Snyder, Euan A. Ashley

**Affiliations:** 1 Department of Genetics, School of Medicine, Stanford University, Stanford, CA USA; 2 Stanford Center for Genomics and Personalized Medicine, Stanford University, Palo Alto, CA USA; 3 Clinical Genomics Program, Stanford Health Care, Stanford, CA USA; 4 Department of Cardiovascular Medicine, Stanford University, Stanford, CA USA; 5 Department of Biomedical Data Science, Stanford University, Stanford, CA USA; 6 Present Address: School of Medicine, Research IT - Technology and Digital Solutions, Stanford University, Redwood City, CA USA; 7 Present Address: Roche Diagnostics Solutions, Research and Early Development, Pleasanton, CA USA

**Keywords:** Benchmarking, Workflow, GIAB reference genomes, Precision, Recall, Truth set, Docker, Germline variants, Lab developed tests

## Abstract

**Background:**

Benchmarking the performance of complex analytical pipelines is an essential part of developing Lab Developed Tests (LDT). Reference samples and benchmark calls published by Genome in a Bottle (GIAB) consortium have enabled the evaluation of analytical methods. The performance of such methods is not uniform across the different genomic regions of interest and variant types. Several benchmarking methods such as hap.py, vcfeval, and vcflib are available to assess the analytical performance characteristics of variant calling algorithms. However, assessing the performance characteristics of an overall LDT assay still requires stringing together several such methods and experienced bioinformaticians to interpret the results. In addition, these methods are dependent on the hardware, operating system and other software libraries, making it impossible to reliably repeat the analytical assessment, when any of the underlying dependencies change in the assay. Here we present a scalable and reproducible, cloud-based benchmarking workflow that is independent of the laboratory and the technician executing the workflow, or the underlying compute hardware used to rapidly and continually assess the performance of LDT assays, across their regions of interest and reportable range, using a broad set of benchmarking samples.

**Results:**

The benchmarking workflow was used to evaluate the performance characteristics for secondary analysis pipelines commonly used by Clinical Genomics laboratories in their LDT assays such as the GATK HaplotypeCaller v3.7 and the SpeedSeq workflow based on FreeBayes v0.9.10. Five reference sample truth sets generated by Genome in a Bottle (GIAB) consortium, six samples from the Personal Genome Project (PGP) and several samples with validated clinically relevant variants from the Centers for Disease Control were used in this work. The performance characteristics were evaluated and compared for multiple reportable ranges, such as whole exome and the clinical exome.

**Conclusions:**

We have implemented a benchmarking workflow for clinical diagnostic laboratories that generates metrics such as specificity, precision and sensitivity for germline SNPs and InDels within a reportable range using whole exome or genome sequencing data. Combining these benchmarking results with validation using known variants of clinical significance in publicly available cell lines, we were able to establish the performance of variant calling pipelines in a clinical setting.

## Background

Next Generation Sequencing (NGS) and analytical methods developed to detect various forms of disease-causing polymorphisms are now routinely being used by clinical laboratories to determine the molecular etiology of complex diseases or disorders and in many cases to make critical treatment course decisions. In the past two decades, many polymorphisms in the human genome have been identified and validated that serve as predictive, diagnostic, and prognostic markers for complex inherited diseases. These genomic disease markers can be of different forms, such as Single Nucleotide Variants (SNVs), small INsertions and DELetions (InDels), large deletions and duplications, and Copy Number Variations (CNVs), and can vary in size from a single base change to several Mega Bases (MB) in length and even whole chromosomal polysomies. Clinically relevant polymorphisms occur in the different regions of the genome, including exonic, splice-sites, and deep-intronic regions. These polymorphisms also happen in various forms, including single base changes within high entropic regions, copy number changes to homopolymer repeats and copy number changes to Short Tandem Repeat (STR) regions. NGS platforms with disparate sequencing chemistries and signal processing methods used to detect these polymorphisms also operate under various error modes; hence, they have very different analytical performances across regions of the genome. Consequently, analytical methods specific to various NGS platforms such as Illumina, Ion Torrent, Pacific Biosciences, and Oxford Nanopore have been developed to both account for and correct the errors particular to these sequencing platforms. A dizzying array of combinations of sequencing platforms and analytical methods are now available to a clinical diagnostic laboratory to develop their LDT assays. Therefore, this presents a challenge to the laboratory staff to determine which combination is the optimal.

To meet this challenge, methods of benchmarking systems and pipelines are used to accurately assess the performance of sequencing platform and analytical method combinations before they are incorporated into a clinical diagnostic assay. Benchmarking starts with a set of data for which the relationship between the input and output is known, so that the sequencing system can be tested to see if, given the same input, it produces the same output, or at least something acceptably close. The Genome In A Bottle (GIAB) consortium hosted by NIST has provided that data for a pilot genome (NA12878/HG001) [1] and for six samples from the Personal Genome Project (PGP) [2]. The established, ground-truth calls for SNVs and small InDels (1–20 base pairs) from these reference samples can be used for optimization, performance estimation, and analytical validation of LDT assays using complex analytical pipelines with multiple methods to detect polymorphisms in the genome. To assist with assessing benchmarking runs, the Global Alliance for Genomics and Health (GA4GH) benchmarking team has developed standardized tools [3] to evaluate the performance metrics of germline variant callers used primarily in research applications.

Results from these types of benchmarking techniques allow a laboratory to demonstrate that its practices meet the exacting standards which certify laboratories for the use of their NGS results in the care of clinical patients. The Clinical Laboratory Improvement Amendments (CLIA) program requires that all laboratories using LDT must establish the test's performance specifications, such as analytical sensitivity, specificity, reportable range, and reference range [4]. The College of American Pathologists (CAP) laboratory standards for NGS based clinical diagnostics [5] not only require the laboratories to assess and document the performance characteristics of all variants within the entire reportable range of LDTs but also obtain the performance characteristics for every type and size of variants that are reported by the assay. Laboratories are also required to assess the performance characteristics for clinically relevant variants, such as $\Delta F508$ and IVS8-5T [6] mutations in a CFTR assay. The CAP guidelines also require laboratories to periodically (determined by the laboratory) assess and document the analytical performance characteristics to ensure that the LDT is continuing to perform as expected over time.

Benchmarking workflows that are highly scalable, reproducible and capable of reporting the performance characteristics using many reference and clinical samples are needed. In addition, evaluation within multiple highly stratified regions of interest are essential for clinical laboratories to optimize and routinely assess the performance of their LDT assays. Jeremy Leipzig in his comprehensive review of bioinformatics workflows for NGS applications [7] defines a bioinformatics workflow as a structured sequence of methods or scripts that are executed either in sequence or parallel to achieve a complex set of analytical goals that are not feasible by any single program. The individual steps within the workflow help achieve a specific goal by accepting a set of inputs and transforming them to a desired set of outputs, which in-turn serve as inputs to other steps later in the workflow. Benchmarking workflows for NGS based clinical assays typically start with variant calls in form of VCF files as inputs to generate desired assay performance characteristics used during the assay development and validation. In order to accomplish this overall objective, workflows deploy widely-used variant comparison programs, such as *vcfeval* [8], *hap.py* [9], *SURVIVOR* [10], *SURVIVOR_ant* [11] and *svclassify* [12], which are capable of variant allele normalization, genotype matching, variant classification and breakpoint matching for structural variants. However, due to ambiguity in allelic representations (especially in cases of adjacent SNPs and InDels) and differing normalization methods employed by each of these programs, the computed performance characteristics can vary depending on the program used in the benchmarking workflows. To overcome this challenge, benchmarking workflows can deploy multiple methods in parallel and either report a consensus-based assessment or report all the performance characteristics that are estimated by the individual methods. Thus, the overall value of a benchmarking workflow is not just in the methods that are included but also in the specific way that they are deployed within the workflow and the reliability of the assay’s overall performance characteristics reported by the workflow.

In addition, benchmarking workflows deployed in evaluation of clinical-diagnostic assays should meet minimum precision guidelines for both repeatability and reproducibility [13]. CLIA guidelines have adopted the International Organization for Standardization definitions of reproducibility and repeatability for clinical assays. Repeatability (within-run) is the measure of precision involving assays carried out under the same experimental conditions, such as reagent lots, instrument, laboratory and operator. Reproducibility (intra-run) is the measure of precision involving assays carried out under different conditions, such as reagent, operator, laboratory, etc. In terms of benchmarking workflows, repeatability is defined as the ability of the workflow to reliably generate the same set of performance metrics given the same set of input variant call files. Though achieving high levels of precision for repeatability might seem trivial for software programs, in-practice, it requires a high degree of engineering to achieve. The workflows should be able to track and detect changes to input files (using MD5 checksums), software libraries, underlying operating systems and even hardware architecture. Also, benchmarking workflows, even individual methods used within these workflows, are often not reproducible as they are often custom developed for a laboratory setting, such as hardware configuration, software versions, and in some cases even the method of execution. The level of effort needed to port these pipelines successfully to another laboratory with different operating conditions is often insurmountable, resulting in a plethora of non-standard workflows with no discernable way to compare the results.

Thus, there is a critical need for benchmarking workflows that can meet the high precision requirements for both reproducibility and repeatability of a clinical assay. These workflows should also be highly scalable to meet the growing adoption of NGS based assays in the clinical diagnostic setting.

## Results

Our goal was to develop a benchmarking workflow that any clinical laboratory could use to quickly evaluate and compare the performance characteristics of all suitable secondary analysis pipelines such as those employing variant callers. A benchmarking workflow should further help optimize the analytical workflow based on well-defined performance metrics and finally produce a thorough analytical validation report to justify the use of the analytical workflow in their diagnostic assay to regulatory authorities such as CLIA and CAP.

To test the abilities of our benchmarking workflow, we used it to compare two analytical workflows commonly used for germline variant calling: (1) workflow based on Broad Institute’s best practices guidelines using the *GATK HaplotypeCaller* v3.7 [14] and (2) the *SpeedSeq* workflow [15] based on *FreeBayes* v0.9.10 [16] as the primary variant calling engine. The GATK HaplotypeCaller based workflow was chosen over the FreeBayes based workflow as it out-performed in the detection of small-InDels (1–20 base pairs). For reference, the benchmarking results for both the above workflows are available for two GIAB samples (NA24143 and NA24149) in the Additional files 1, 2, 3, 4: Tables S1–S4. In addition, the comparison between the InDel size distribution numbers for the GATK HaplotypeCaller and SpeedSeq workflows for one GIAB sample (NA24631) is presented in the Additional file 5: Table S5.

The performance characteristics of the analytical workflow using GATK v3.7 was further optimized using benchmarking metrics generated from the five GIAB reference samples and four GeT-RM samples (see “Methods” section) with known pathogenic variants. Also, it is critical for the clinical laboratories developing NGS based LDT assays to accurately determine the reportable range to avoid misdiagnosis which would lead to wrong treatment decisions. To this end, we evaluated the performance metrics using the benchmarking workflow in three distinct genomic regions of interest (see “Methods” section for details).

Although we have the benchmarking results for the region including coding exons in all the RefSeq genes, we have omitted those findings in this section and focus only on the clinically relevant regions.

Tables 1 and 2 show the benchmarking metrics for SNPs in all 5 GIAB samples within the clinically relevant genes and whole exome regions, respectively. The precision, recall, and negative percent agreement (NPA) metrics for SNPs are uniform across all the reference samples, and there is no sample bias in results for some of the better-characterized samples such as NA24385 and NA12878. Performance metrics for SNPs within the clinically relevant gene region are significantly better than those within the whole exome region. It is notable that recall metrics are a percentage point better in the clinically pertinent gene region, across all reference samples. This phenomenon is attributable to the fact that many genes have isoforms, resulting in higher alignment errors, and some genes have either very high or very low GC content, resulting in higher than average sequencing errors within these regions of the genome. This finding is of great clinical significance, since the reportable region of most inherited disease or disorder-diagnostic based LDT assays are limited to the clinically relevant genes. Thereby, the overall performance characteristics of the assay is better than that estimated over either the whole genome or whole exome regions.

**Table 1:** Benchmarking metrics for SNPs within coding exons of ~ 7000 clinically relevant genes (as specified in “Methods” section)

GIAB genome/NIST ID	Number of bases	Truth total	TP	FP	FN	TN	NPA	Precision	Recall
NA12878	13,728,555	7803	7781	4	22	13,720,748	100	99.95	99.72
NA24143	12,549,224	7470	7460	14	10	12,541,740	100	99.81	99.87
NA24149	12,538,042	7495	7485	19	9	12,530,529	100	99.75	99.88
NA24385	12,626,866	7452	7436	0	16	12,619,414	100	100	99.79
NA24631	12,808,688	7591	7581	6	10	12,801,091	100	99.92	99.87

**Table 2:** Benchmarking metrics for SNPs in whole exome regions, including non-coding exons, splice sites (± 20 bp) and clinically relevant deep intronic regions

GIAB genome/NIST ID	Number of bases	Truth total	TP	FP	FN	TN	NPA	Precision	Recall
NA12878	71,152,019	57,822	57,024	491	776	71,093,728	100	99.15	98.66
NA24143	65,657,646	55,975	55,340	669	611	65,601,026	100	98.81	98.91
NA24149	65,597,266	55,518	54,827	669	669	65,541,101	100	98.79	98.79
NA24385	65,948,744	56,068	55,329	389	705	65,892,321	100	99.30	98.74
NA24631	66,988,987	56,948	56,303	394	643	66,931,647	100	99.31	98.87

Tables 3 and 4 provide the InDel benchmarking metrics for sample NA24385 in the clinically relevant and whole exome regions, respectively. As expected, the benchmarking workflow reveals that the performance metrics for InDels are lower than those for SNPs. However, the stratification by InDel size, helped us determine the reference range for InDels (1–20 base pairs). The recall metric for InDels larger than 20 base pairs is significantly lower than the recall for InDels 1–20 base pairs. As in the case of SNPs, performance metrics for InDel detection within the clinically relevant genes of interest is better than the whole exome region.

**Table 3:** Benchmarking metrics for InDels of different size ranges in NA24385 (truth set NIST v3.3.2, total bases = 12,626,866) for the regions within ~ 7000 clinically relevant genes (as specified in “Methods” section)

Size of InDels in NA24385	Truth total	TP	FP	FN	TN	NPA	Precision	Recall
1–10	145	136	12	9	12,626,709	100	91.89	93.79
11–20	9	9	0	0	12,626,857	100	100	100
21–50	3	3	0	0	12,626,863	100	100	100
All Indels	157	148	12	9	12,626,697	100	92.50	94.27

**Table 4:** Benchmarking metrics on the number of InDels of different size ranges in NA24385 (truth set NIST v3.3, total bases = 65,948,744) for the whole exome regions including non-coding exons, splice sites (± 20 bp) and clinically relevant deep intronic regions

Size of InDels in NA24385	Truth total	TP	FP	FN	TN	NPA	Precision	Recall
1–10	5169	4727	872	442	65,942,703	100	84.43	91.45
11–20	203	188	10	15	65,948,531	100	94.95	92.61
21–50	67	56	3	11	65,948,674	100	94.92	83.58
All Indels	5362	4920	885	468	65,942,471	100	84.75	91.27

The benchmarking results of the other GIAB reference samples in the clinically relevant and whole exome regions can be obtained in the Additional files 6, 7, 8, 9: Tables S6–S9 and Additional files 10, 11, 12, 13: Tables S10–S13, respectively. The histograms for the InDel size distribution in the NA24385 reference sample for the clinically relevant and whole exome regions respectively are in Additional file 17: Fig. S1. The histograms of InDel size distributions for GIAB samples in both the whole exome and clinically relevant regions are available in the github repository—*StanfordBioinformatics/stanford-benchmarking-workflows*.

Finally, the benchmarking workflow was able to confirm that the variant calling pipeline can detect all the clinical variants in GeT-RM samples listed in Table 5.

**Table 5:** Validation of the presence of the truth variants in the GeT-RM samples with their locations specified as GRCh37 coordinates used in our variant calling workflow

GeT-RM sample ID	Chromosome: position	Truth variant	Truth variant detected
NA04408	15: 91,310,152	TATC → T	Yes
	15: 91,310,156	T → TA	Yes
	15: 91,310,158	A → ATTC	Yes
NA14090	17:41,276,044	ACT → A	Yes
NA14170	13:32,914,437	GT → G	Yes
NA16658	10:43,609,103	G → T	Yes

To obtain all the metrics produced by hap.py and other output files including InDel size distribution plots from our benchmarking workflow for each reference sample, please refer to our GitHub repository.

Additionally, we generated benchmarking metrics and ROC curves for NA24143 using a tool provided by Real Time Genomics (RTG) [8]. The results for benchmarking in the clinically relevant regions and details on the metrics for the other two regions of interest can be found in Additional files 14, 15, 16: Tables S14–S16. Similarly, Additional files 18, 19, 20: Figs. S2–S4.

## Discussion

The GIAB consortium has helped develop standards for genomic data to evaluate the performance of NGS sequencing platforms and analytical methods used for alignment and variant calling. The precisionFDA platform [17] has enabled the genomics community to develop and deploy benchmarking methods that can evaluate the performance of analytical methods against the gold standard datasets, such as ones made available by GIAB. These benchmarking tools, along with accuracy challenges, have led to the development of highly accurate variant calling methods. However, the requirements of a clinical diagnostic laboratory go beyond the simple evaluation of performance characteristics of an analytical pipeline against one or more reference samples. Our purpose was to build a benchmarking workflow to meet the assay optimization and validation needs of a clinical laboratory. The primary benefit of our benchmarking workflow is that it allows for the assay performance to be evaluated using a broad set of both reference samples with a large number of gold-standard variant calls and clinical samples with a small number of clinical variants that are specific to the diagnostic assay being evaluated. The benchmarking workflow enables the clinical laboratories to establish the reporting range of the diagnostic assay by estimating the performance within multiple regions of interest.

The precisionFDA platform has enabled a benchmarking pipeline (vcf comparison framework) which accepts input and truth set variant call files and regions of interest files in BED format to calculate the assay performance metrics across the entire region of interest. The vcf comparison pipeline is only capable of comparing variant calls one sample at a time. The Association for Clinical Genomic Science [18] has published a cloud-based benchmarking pipeline similar to the precisionFDA pipeline. However, this pipeline is more restrictive than the precisionFDA pipeline in that it is capable of evaluating the performance using only one (NA12878) of the several benchmarking samples published by GA4GH. The Seven Bridges platform recently published another cloud-based benchmarking pipeline [19] capable of evaluating the performance characteristics using several GA4GH benchmarking samples and multiple regions of interest at the same time. All the cloud-based benchmarking workflows require the clinical laboratories to upload their sensitive assay results, which in some cases include germline variant calls from WGS assays, to a public cloud platform which may not be HIPAA compliant.

Unlike these web-based benchmarking apps such as those provided by the precisionFDA platform or GA4GH, our benchmarking framework can be seamlessly integrated with any variant calling pipeline in the user's software environment. Thus, our benchmarking workflow enables ease of use and avoids the transfer of sensitive data to different locations.

The emphasis on repeatability and reproducibility by building containerized solutions in clinical settings are starting to emerge in other fields, such as medical imaging and neurosciences [20, 21]. Similarly, the benchmarking workflow is able to achieve high precision for both repeatability and reproducibility by being agnostic to the hardware infrastructure used to execute it. The individual software modules within the workflow are deployed as Docker [22, 23] containers which are self-contained with all the prerequisite software libraries and other dependencies. The Docker images of individual software modules have been published to a public container repository, Docker Hub [24].

The benchmarking workflow is distributed using human-readable YAML [25] format, and it can be ported to existing WDL based workflows, which are executed using workflow managers like Cromwell, published by the Broad Institute [26, 27]. Similarly, the workflow YAML files can be ported to the Common Workflow Language (CWL) format [28, 29] to use pipeline execution engines published by the GA4GH [30]. The workflow can be readily deployed by the clinical laboratories either within their on-premises computer infrastructure, private-cloud or any of the available public-cloud platforms, such as the Google Cloud Platform, Amazon Web Services and Microsoft Azure.

Our benchmarking modules integrated with deployment tools, such as Jenkins [31] or CircleCI [32], that work on the principle of continuous integration and continuous delivery/deployment (CI/CD) can provide a fool proof way of examining consistency in results. In this era where workflows generating reproducible results are gaining attention, easy incorporation of workflows with CI/CD tools is a nice feature to have.

## Conclusions

Benchmarking variants is a critical part of implementing variant calling pipelines for research or clinical purposes. Here, we have successfully implemented a benchmarking workflow that generates metrics, such as specificity, precision and sensitivity for germline SNPs, and InDels in whole exome sequencing data. Also, InDel size distributions even in the form of histograms are also provided. Moreover, the parameters within each tool and predefined InDel size bins can be easily modified in the benchmarking workflow to suit a laboratory’s requirements. Combining these benchmarking results with validation using known variants of clinical significance in publicly available cell lines, we were able to establish our variant calling pipelines in a clinical setting. Our benchmarking workflow can serve as a plug-in to any existing variant calling pipeline to work as an integrated unit or be used as a separate module as well.

Furthermore, future extensions to the existing benchmarking workflow can be made to accommodate automatic generation of benchmarking metrics and ROC curves from other tools such as RTG for which we have the public Docker image readily available.

A benchmarking workflow similar to the one built in this work for benchmarking short variants can be constructed to perform benchmarking of structural variants (SVs).

## Methods

### Benchmarking workflow

**Fig. 1:**
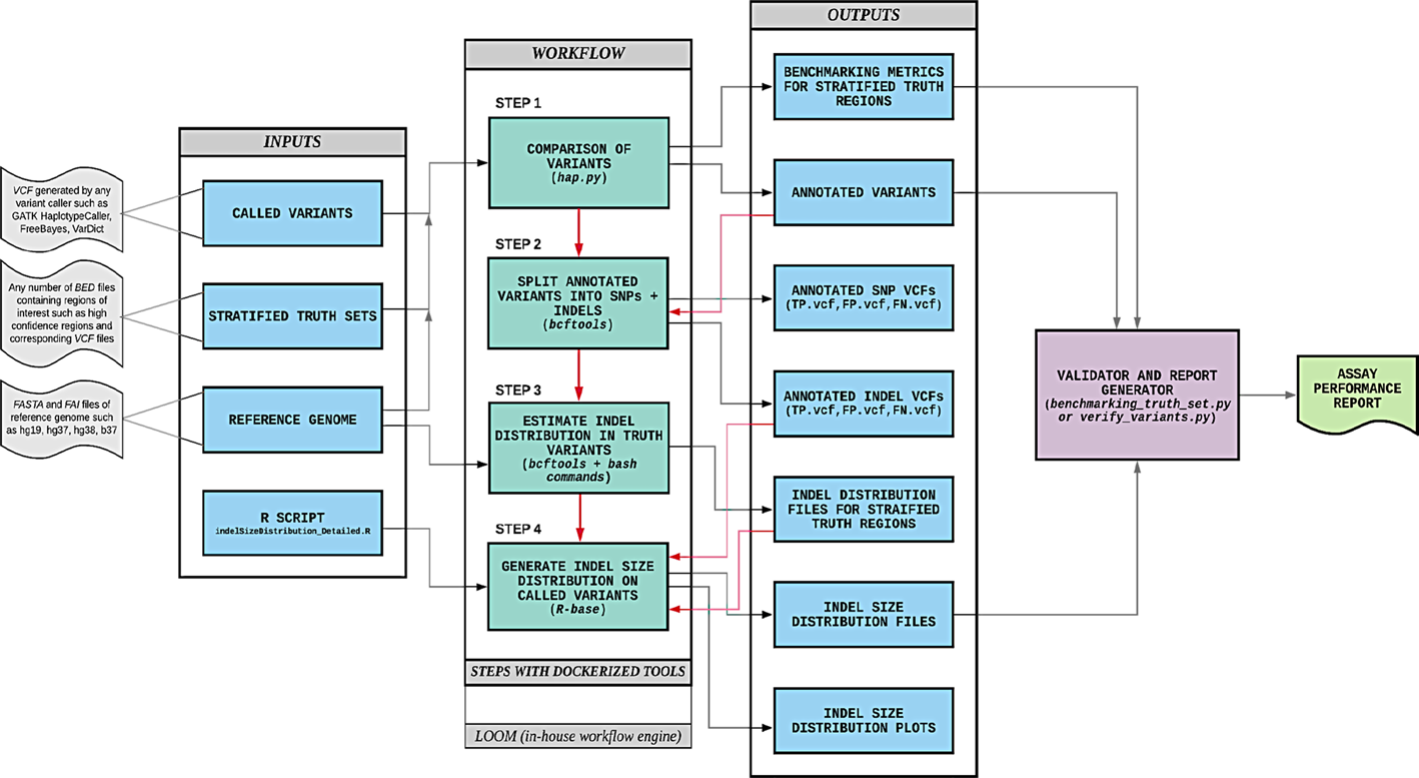
Schematic diagram of the benchmarking framework used in this study. The benchmarking workflow written in YAML format consists of four main steps in which the software tool employed in each step is dockerized and indicated within parentheses. The details of the Docker images for these software tools, which are available in Docker Hub, are specified in the “Methods” section. This benchmarking workflow was executed using Loom, an in-house workflow engine. The inputs to the benchmarking workflow are depicted on the left and the outputs are on the right with the red arrows from the outputs to workflow steps indicating the dependency of the output of that step with another step. The steps in the benchmarking workflow are repeated for each stratified region of interest provided

The benchmarking workflow, as illustrated in Fig. 1, is a sequence of steps required to perform a rapid and comprehensive analytical validation of a clinical diagnostic assay based on germline variants. The benchmarking workflow can be easily integrated with any secondary-analysis pipeline used in a diagnostic assay to call germline variants, and the workflow accepts germline variants (SNVs and small InDels) in Variant Call Format VCF v4.1 [33] or higher. The workflow takes one or more stratification files specifying the regions of interest in BED [34] format and generates a comprehensive analytical validation report detailing the performance characteristics of the assay within each of the specified regions of interest. The benchmark variant calls that are considered as ground truths for each of the reference samples used to evaluate the analytical performance can also be specified in VCF format.

**Fig. 2:**
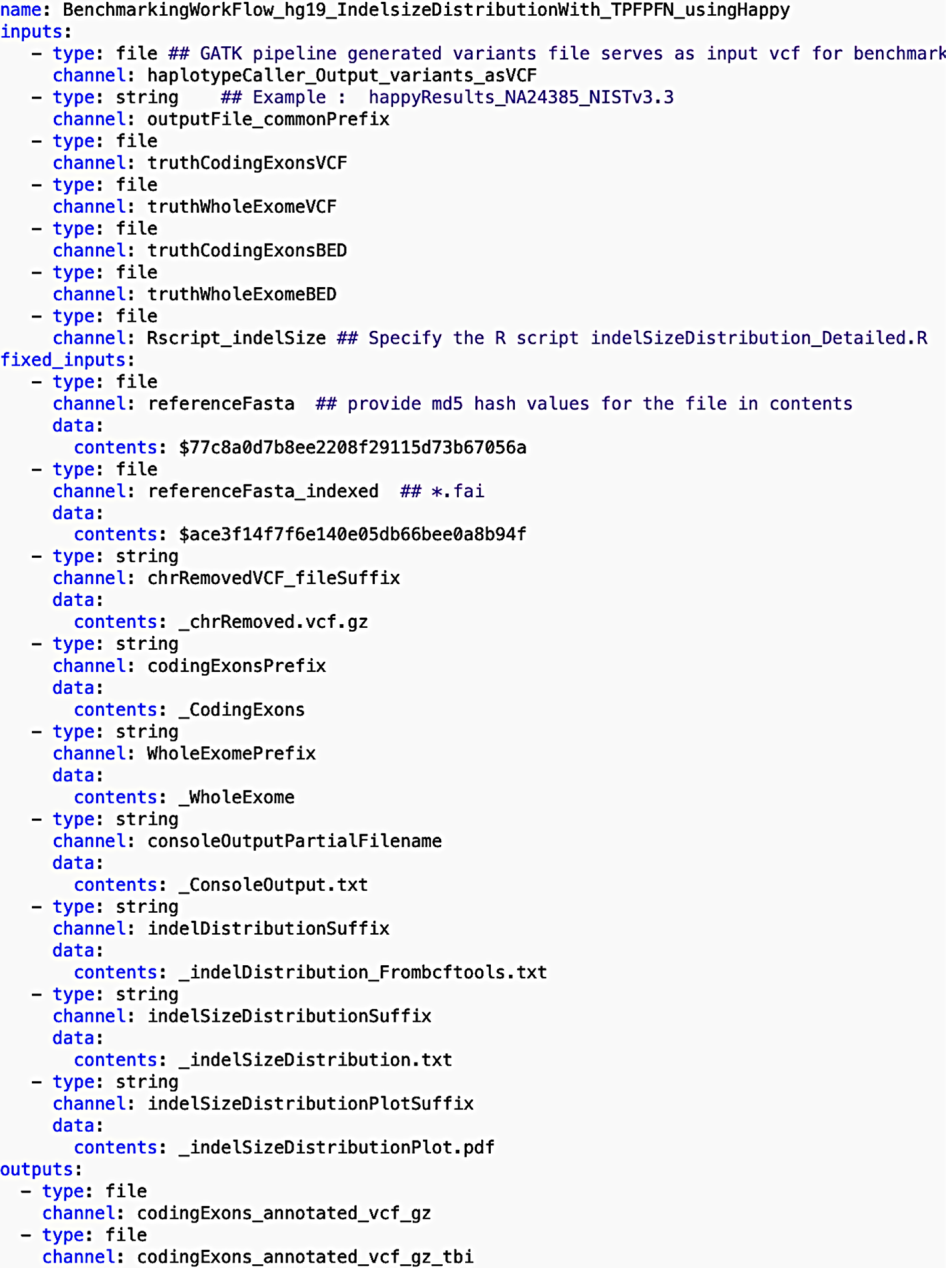
Snippet of the benchmarking.yaml depicting the structure of the beginning of the benchmarking workflow before the steps are defined

The basic structure of the benchmarking workflow listing all the initial inputs and outputs expected from all the steps before the individual steps are defined is shown in Fig. 2. The name of the workflow is specified first followed by the entire list of variable input files required for the workflow. Next, we listed inputs that are fixed, which can be files or strings required in any step in the workflow. These “fixed_inputs” are those that do not change often even when the workflow is run multiple times, such as the reference fasta file and its corresponding index file in this case. For each “fixed_input”, the sub-field “data” has “contents” which is assigned a value based on “type” provided. Depending on the “type” specified for a certain input, the “contents” value is interpreted differently. For a “string” type, “contents” contains a text value. For a “file” type, “contents” contains a unique identifier such as “[filename]$ [MD5]”. Please note that “fixed_inputs” is just a term we adopted in order to segregate infrequently changed inputs from variable inputs, such as different regions of interest or ground-truth VCF files. The user can modify or remove the files or strings in the fixed input category as required, and it does not affect the functionality or mode of execution of the workflow. Subsequently, we listed the entire list of output files generated by all the steps in the workflow including intermediate files. In this YAML structure of workflow, the “type” of input or output is specified for example as a string or file. A “channel” refers to a designed name (similar to a variable name in a script) for a particular input or output that the workflow manager uses during execution.

**Fig. 3:**
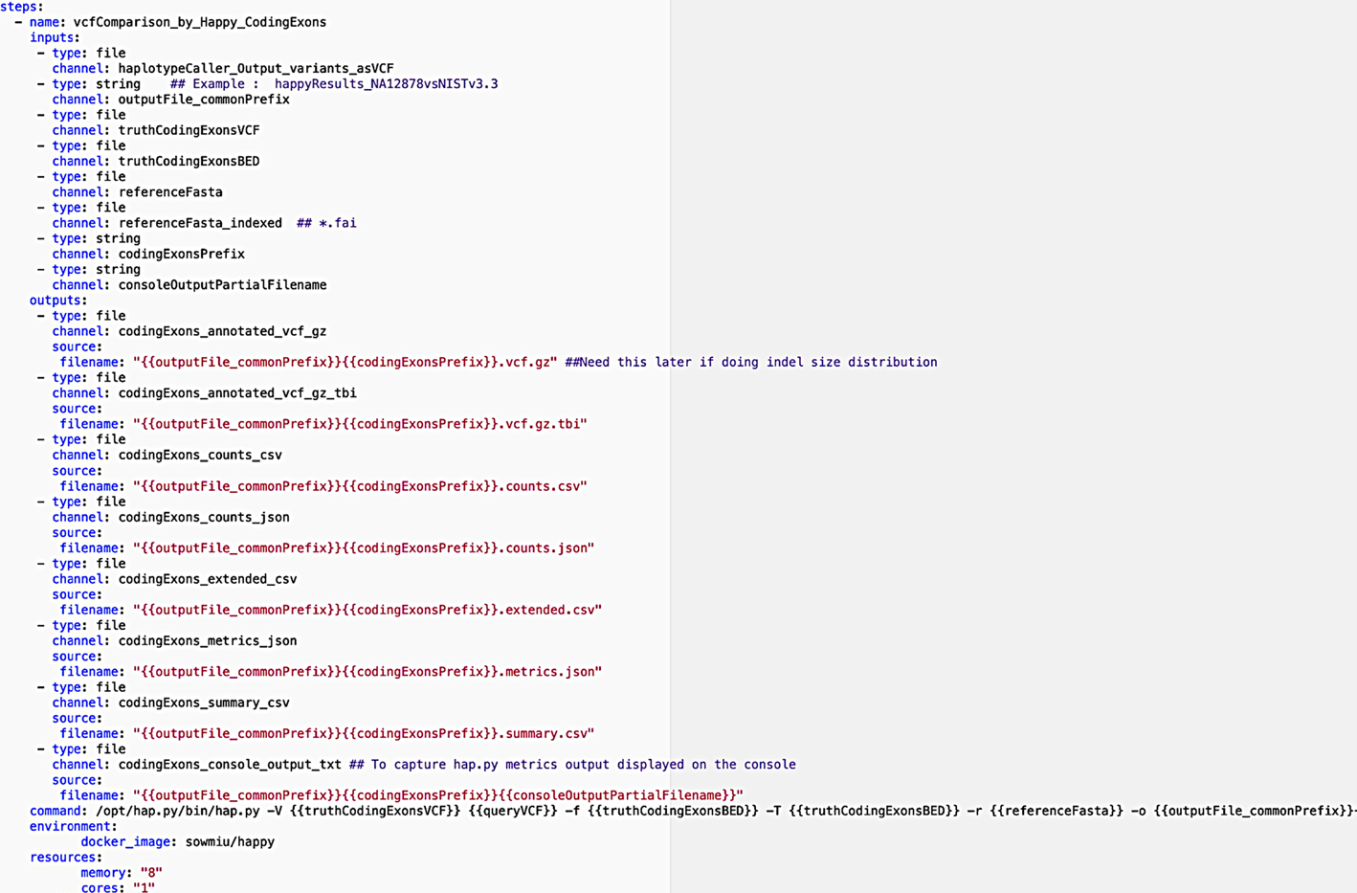
Snippet of the benchmarking.yaml showing the first step (vcf comparison using hap.py) of the benchmarking workflow

A typical structure of a step in a workflow is presented in Fig. 3, where the “steps” tag denotes the beginning of all the steps listed in that workflow. A step begins with the name of the step followed by the “inputs” and “outputs” pertaining only to that step. Here, aside from “type” and “channel”, an output has a “source” tag specified in the form of a “key:value” pair where the key is “filename”. The value for “filename” is a string that can be a “channel” from “fixed_inputs” or an aggregation of different “channels” listed in the “fixed_inputs”, which can be optionally concatenated with a user defined string. The “command” tag specifies the command line consisting of the software tool, parameters, and the inputs and outputs to be executed inside the docker container. The “environment” tag contains a “key:value” pair in the form of “docker_image” as the key and the value as the Docker image name required for that step. The Docker image can be pulled from Docker Hub or the Docker container registry in the user’s computing platform. The compute resources to be utilized for a workflow step is provided via the “resources” tag containing two “key:value” pairs where “memory” and “cores” are the keys whose values are strings denoting memory in GB and number of cores respectively.

The first step in the benchmarking process as seen in Fig. 3 involves the comparison of input variants generated by the analytical pipeline with the benchmark variant calls within each region of interest. The variant calls are compared using hap.py, which is capable of haplotype construction from individual genotype calls and is recommended by GIAB consortium and GA4GH. The variant comparison step is performed for each stratification or region of interest file specified as input, and hap.py generates a single output VCF file classifying the variant calls defined in the input and truth VCF files as either True Positive (TP), False Positive (FP) or False Negative (FN).

Step two in the benchmarking workflow splits the variant calls annotated using hap.py by variant type (SNPs and small InDels) and by variant classification (TP or FP or FN). This step is executed within the workflow for each of the stratification or region of interest files specified. The VCF files are split by variant type using *bcftools* [35], and a bash script is used to further split the variant calls by the variant classification. This allows the workflow to generate the performance metrics for each of the variant types reported by the diagnostic assay.

Steps three and four of the benchmarking workflow (see Fig. 1) were used to generate a histogram of small InDels by size. The bins used for InDel size histograms were (a) 1–10 base pairs, (b) 11–20 base pairs, (c) 21–50 base pairs, and (d) Greater than 50 base pairs. The R script—*indelSizeDistribution_Detailed.R* (code in Additional file 21: File 1) then calculates the performance metrics of the assay for each of the InDel size bins. The Python script—*benchmarking_truth_set.py* (Additional file 22: File 2) consolidates the benchmarking metrics previously obtained and calculates the NPA related metrics combining some of the bin size ranges (user preferred) for all reference samples provided. The details on the usage of the above script are in the associated README file available in our public repository.

In addition to benchmarking truth sets for well-characterized reference samples published by the GIAB consortium, the benchmarking workflow allows clinical laboratories to specify additional samples with clinically relevant variants as ground-truths to estimate the analytical performance of the assay for specific variant types, such as $\Delta F508$ and IVS8-5 T in CFTR panels. The Python script—*verify_variants.py* (Additional file 23: File 3) accepts the ground-truth variant call sets to confirm the presence or absence of these variants in the VCF files generated by the variant calling pipeline.

Finally, the benchmarking workflow generates a comprehensive analytical validation report using all the provided benchmarking ground-truth call sets—*Final_benchmarking_metrics.txt* (Additional file 24: File 4).

### Scalability and reproducibility of benchmarking workflow

The benchmarking workflow is designed to be repeatable and reproducible by using Docker containers for all software and bioinformatics components used within the workflow (see Table 6). The workflow is distributed in a human-readable data serialization format YAML v1.2, and the workflow can be readily executed using the workflow execution manager—*Loom* (0.5.3-prerelease-rc10) [36]. The workflow definition file—*Benchmarking.yaml* (see our GitHub repository) can also be easily ported to Common Workflow Language (CWL) or Workflow Definition Language (WDL) formats and executed using workflow execution managers, such as Toil [37, 38] and Cromwell.

**Table 6:** List of software components utilized in the benchmarking workflow with their software dependencies, settings and Docker image names (as available in the Docker Hub repository) if applicable

Software component	Docker image	Other software dependencies and settings included
hap.py v0.2.10	sowmiu/happy	Ubuntu 14.04
		Python 2.7.6, python2.7-dev, python-software-properties, cython, numpy, pandas, setuptools, pybedtools, pysam, bx-python, nose, pip, numpy, Distribute
		Cmake > 2.8, gcc/g++4.8 + zlib1g-dev, libncurses5-dev, bzip2, wget, libbz2-dev, build-essential, libatlas-base-dev, pkg-config, boost 1.55 + , software-properties-common
		git, samtools 0.1.19, bcftools 0.1.19, gfortran
bcftools	vandhanak/bcftools:1.3.1	Ubuntu 14.04
		Make, g++, gcc, zlib1g-dev, libgsl0ldbl, gsl-bin, libgsl0-dev, libatlas-base-dev git, htslib 1.3.2
IndelSizeDistribution_Detailed.R	vandhanak/rbase:3.3.2	Ubuntu 14.04
		libcurl4-openssl-dev, libxml2-dev, locale setting: en_US.UTF-8
		Set access to these repositories: trusty-backports, CRAN
Benchmarking_truth_set.py	–	Python 2.7
Verify_variants.py	–	Python 2.7

### Golden/ground-truth call sets

The golden/ground-truth sets for five reference and PGP genomes are currently available—NA12878 (CEPH family's daughter), NA24143 (AJ mother), NA24149 (AJ father), NA24385 (AJ son) and NA24631 (Chinese son), and these reference call sets were used in this benchmarking study. GIAB provides a high confidence regions file and a high confidence VCF file, and as recommended by GIAB, only the high confidence calls were used in the evaluation of the assay's performance characteristics. The NIST versions and their corresponding FTP site locations used for the above samples in this study can be found in the Additional file 25.

In addition to the GIAB reference samples, samples with known pathogenic germline variants (see Table 5) for various inherited diseases or disorders were chosen from the Genetic Testing Reference Materials Coordination Program (GeT-RM) [39–43].

### Stratification or regions of interest (ROI) BED files.

Three stratification files were used to evaluate the performance characteristics of an inherited Whole Exome Sequencing (WES) assay.

1. Coding Exons for all known transcripts in RefSeq genes: RefSeq gene names, transcripts, and coordinates of all coding exons were obtained from the UCSC genome browser [44–46].
2. Clinically relevant regions of the human genome: Clinically relevant regions were determined by intersecting coordinates of all known pathogenic variants reported in OMIM [47], ClinVar [48] and DECIPHER v9.28 [49] with all the exon regions (coding and non-coding) file for RefSeq genes obtained from the UCSC genome browser. The exonic coordinates were later extended by 20 base pairs on either end to include canonical and non-canonical splice sites. Deep-intronic regions with pathogenic variants were added to the exonic regions to generate the final clinically relevant regions (BED) file.
3. Whole Exome regions file for RefSeq genes: Whole Exome regions file was obtained from the UCSC genome browser. The exon regions were extended by 20 base pairs on either end to include splice sites.

### Benchmarking metrics

Precision and recall are benchmarking metrics provided as output by hap.py. The true positives (TP), false positives (FP), and false negatives (FN) are counted as described by the developers of hap.py. Again, as explained by the authors of hap.py, precision and recall are calculated using the below formulae:

$$\begin{aligned} Precision \, & = \, True \, \,Positives/\left( {True\, \, Positives \, + \, False\, \, Positives} \right) \\ Recall \, & = \, True \, \,Positives/\left( {True \, \,Positives \, + \, False \, \,Negatives} \right) \\ \end{aligned}$$

Other metrics reported by hap.py, such as variants outside the high confidence truth set regions and transition or transversion SNP type, can be found in the extended.csv files included in the vcfComparison directories for each GIAB sample in our GitHub repository..

The total number of bases per sample in a particular region of interest as specified by the corresponding bed file was computed using a bash command provided in the Additional file 25.

True negatives (TN) and Total Negatives are computed using the following:

$$\begin{aligned} & TN \, = \, Total\, \, number\, \, of \, \,bases \, \,in \, \,the\, \, region \, \,of\, \, interest \, - \, \left( {True \, \,Positives \, + \, False\, \, Positives \, + \, False \, \,Negatives} \right) \\ & Total \, Negatives \, = \, True \, \,Negatives \, + \, False \, Positives \\ \end{aligned}$$

The Negative Percentage Agreement (NPA) or specificity as recommended by the FDA [50] is calculated using the following:

$${\text{NPA }} = True \, \,Negatives/Total \, \,Negatives.$$

### Generation of ROC curves outside of benchmarking workflow

There is an option for the user to generate benchmarking metrics and corresponding ROC curves using another popular vcf comparison tool called *vcfeval* in RTG-core’s suite of tools [51]. However, this option is currently not available as part of the validated benchmarking workflow. The ROC plots were obtained using the dockerized version of RTG-core, available in Docker Hub as *vandhanak/rtg-core:3.11*. The input sample VCF used contained variants called by the germline variant caller GATK HaplotypeCaller v3.7. The default value, genotype quality (GQ), for the parameter *vcf-score-field* was used. Further, the parameter *evaluation-regions* was set to the truth bed file corresponding to the region of interest for the GIAB sample. ROC plots were generated using the *rocplot* tool within RTG. In order to demonstrate this functionality, one GIAB sample, NA24143 was used for the benchmarking on the three stratified regions of interest utilizing the corresponding ground-truth sets.

## Supplementary Information

**Additional file 1: Table S1**. Benchmarking metrics on SNPs and multiple nucleotide polymorphisms (MNPs) in NA24149 (truth set NIST v3.3) for the RefSeq coding exons regions generated for both the GATK and SpeedSeq pipelines that were executed using workflows run by Loom (in-house workflow engine).

**Additional file 2: Table S2**. Benchmarking metrics on InDels in NA24149 (truth set NIST v3.3) for the RefSeq coding exons regions generated for both the GATK and SpeedSeq pipelines that were executed using workflows run by Loom (in-house workflow engine).

**Additional file 3: Table S3**. Benchmarking metrics on SNPs and multiple nucleotide polymorphisms (MNPs) in NA24143 (truth set NIST v3.3) for the RefSeq coding exons regions generated for both the GATK and SpeedSeq pipelines that were executed using workflows run by Loom (in-house workflow engine).

**Additional file 4: Table S4**. Benchmarking metrics on InDels in NA24143 (truth set NIST v3.3) for the RefSeq coding exons regions generated for both the GATK and SpeedSeq pipelines that were executed using workflows run by Loom (in-house workflow engine).

**Additional file 5: Table S5**. Benchmarking metrics on InDel size distribution in NA24631 (truth set NIST v3.3.2) for whole exome regions, including non-coding exons, splice sites (+/- 2 bp) and clinically relevant deep intronic regions intersected with clinical exome to assess performance of GATK and SpeedSeq pipelines.

**Additional file 6: Table S6**. Benchmarking metrics for InDels of different size ranges in NA12878 (truth set NIST v3.3, total bases = 13728555) for the regions within ~7000 clinically relevant genes (as specified in Methods).

**Additional file 7: Table S7**. Benchmarking metrics for InDels of different size ranges in NA24143 (truth set NIST v3.3, total bases = 12549224) for the regions within ~7000 clinically relevant genes (as specified in Methods).

**Additional file 8: Table S8**. Benchmarking metrics for InDels of different size ranges in NA24149 (truth set NIST v3.3, total bases = 12538042) for the regions within ~7000 clinically relevant genes (as specified in Methods).

**Additional file 9: Table S9**. Benchmarking metrics for InDels of different size ranges in NA24631 (truth set NIST v3.3, total bases = 12808688) for the regions within ~7000 clinically relevant genes (as specified in Methods).

**Additional file 10: Table S10**. Benchmarking metrics on the number of InDels of different size ranges in NA12878 (truth set NIST v3.3, total bases = 71152019) for the whole exome regions including non–coding exons, splice sites (+/- 20 bp) and clinically relevant deep intronic regions.

**Additional file 11: Table S11**. Benchmarking metrics on the number of InDels of different size ranges in NA24143 (truth set NIST v3.3, total bases = 65657646) for the whole exome regions including non-coding exons, splice sites (+/- 20 bp) and clinically relevant deep intronic regions.

**Additional file 12: Table S12**. Benchmarking metrics on the number of InDels of different size ranges in NA24149 (truth set NIST v3.3, total bases = 65597266) for the whole exome regions including non-coding exons, splice sites (+/- 20 bp) and clinically relevant deep intronic regions.

**Additional file 13: Table S13**. Benchmarking metrics on the number of InDels of different size ranges in NA24631 (truth set NIST v3.3, total bases = 65657646) for the whole exome regions including non-coding exons, splice sites (+/- 20 bp) and clinically relevant deep intronic regions.

**Additional file 14: Table S14**. Benchmarking metrics for NA24143 (SNPs and InDels, truth set NIST v3.3) within coding exons of ~7000 clinically relevant genes (as specified in Methods) using RTG vcfeval.

**Additional file 15: Table S15**. Benchmarking metrics for NA24143 (SNPs and InDels, truth set NIST v3.3) in whole exome regions, including non-coding exons, splice sites (+/- 20 bp) and clinically relevant deep intronic regions using RTG vcfeval.

**Additional file 16: Table S16**. Benchmarking metrics for NA24143 (SNPs and InDels, truth set NIST v3.3) in the RefSeq coding exon regions using RTG vcfeval.

**Additional file 17: Fig S1**. InDel size distribution histograms for NA24385 as generated by the benchmarking workflow for the coding exons of ~7000 clinically relevant genes and whole exome regions (as specified in Methods).

**Additional file 18: Fig S2**. ROC curves for NA24143 within coding exons of ~7000 clinically relevant genes using RTG rocplot with metrics obtained from RTG vcfeval.

**Additional file 19: Fig S3**. ROC curves for NA24143 in the whole exome regions (as specified in Methods) using RTG rocplot with metrics obtained from RTG vcfeval.

**Additional file 20: Fig S4**. ROC curves for NA24143 in the coding exon regions (as specified in Methods) using RTG rocplot with metrics obtained from RTG vcfeval.

**Additional file 21: File 1**. indelSizeDistribution_Detailed.R.

**Additional file 22: File 2**. benchmarking_truth_set.py.

**Additional file 23: File 3**. verify_variants.py.

**Additional file 24: File 4**. Final_benchmarking_metrics.txt.

**Additional file 25**. Supplementary information.

## Data Availability

The benchmarking workflow (benchmarking.yaml), analytical methods or scripts, and datasets generated and analyzed during this study are available in the GitHub repository—https://github.com/StanfordBioinformatics/stanford-benchmarking-workflows.git.

## **List of abbreviations**

NIST: National Institute of Standards and Technology
GIAB: Genome In A Bottle consortium
CFTR: Cystic Fibrosis Transmembrane conductance Regulator
SNPs: Single Nucleotide Polymorphisms
MNPs: Multiple Nucleotide Polymorphisms
InDels: Insertions/Deletions
WES: Whole Exome Sequencing
NPA: Negative Percent Agreement
TN: True Negative
TP: True Positive
FN: False Negative
FP: False Positive
HIPAA: Health Insurance Portability and Accountability Act
OMIM: Public database containing the human genes, their genetic phenotypes, and their associations with genetic disorders (Online Mendelian Inheritance in Man)
DECIPHER: Public database with genotypic and phenotypic data from ~ 30,000 individuals
ClinVar: Public database with information on the relationship between medically important variants and phenotypes.
WDL: Workflow Definition Language
CWL: Common Workflow Language

## Availability and requirements

Project Name: Clinical benchmarking workflow

Project home page: https://github.com/StanfordBioinformatics/stanford-benchmarking-workflows.git

Operating system: Platform independent

Programming language: Python 2.x

Other requirements: Docker, R

License: MIT license

Any restrictions to use by non-academics: None
