# Supplementary material for: Benchmarking workflows to assess performance and suitability of germline variant calling pipelines in clinical diagnostic assays
Source: BMC Bioinformatics. 2021 Feb 24;22:85. doi: 10.1186/s12859-020-03934-3 (PMC7903625; doi:10.1186/s12859-020-03934-3)
Supplement: Supplementary file 2 — Additional file 2: Table S2. Benchmarking metrics on InDels in NA24149 (truth set NIST v3.3) for the RefSeq coding exons regions generated for both the GATK and SpeedSeq pipelines that were executed using workflows run by Loom (in-house workflow engine). [file 12859_2020_3934_MOESM2_ESM.docx]

Additional file 2: Table S2. Benchmarking metrics on InDels in NA24149 (truth set NIST v3.3) for the RefSeq coding exons regions generated for both the GATK and SpeedSeq pipelines that were executed using workflows run by Loom (in-house workflow engine).

| **Workflow run using Loom** | **TP** | **FP** | **FN** | **Precision** | **Recall** |
| --- | --- | --- | --- | --- | --- |
| GATK HaploypeCaller (Broad’s best practices pipeline) | 402 | 27 | 13 | 93.70 | 96.84 |
| SpeedSeq | 367 | 17 | 45 | 95.57 | 89.07 |
