# Supplementary material for: Benchmarking workflows to assess performance and suitability of germline variant calling pipelines in clinical diagnostic assays
Source: BMC Bioinformatics. 2021 Feb 24;22:85. doi: 10.1186/s12859-020-03934-3 (PMC7903625; doi:10.1186/s12859-020-03934-3)
Supplement: Supplementary file 5 — Additional file 5: Table S5. Benchmarking metrics on InDel size distribution in NA24631 (truth set NIST v3.3.2) for whole exome regions, including non-coding exons, splice sites (+/- 2 bp) and clinically relevant deep intronic regions intersected with clinical exome to assess performance of GATK and SpeedSeq pipelines. [file 12859_2020_3934_MOESM5_ESM.docx]

Additional file 5: Table S5. Benchmarking metrics on InDel size distribution in NA24631 (truth set NIST v3.3.2) for whole exome regions, including non-coding exons, splice sites (+/- 2 bp) and clinically relevant deep intronic regions intersected with clinical exome to assess performance of GATK and SpeedSeq pipelines.

| **Indel size** | **GATK HaploypeCaller**  **(Broad’s best practices pipeline)** | | | | **SpeedSeq** | | | |
| --- | --- | --- | --- | --- | --- | --- | --- | --- |
|  | TP | FP | FN | Recall | TP | FP | FN | Recall |
| 1 | 1049 | 120 | 78 | 93.07 | 1065 | 32 | 68 | 93.99 |
| 2–5 | 858 | 112 | 78 | 91.66 | 803 | 51 | 133 | 85.79 |
| 6–10 | 194 | 10 | 7 | 96.51 | 158 | 17 | 48 | 76.69 |
| 11–20 | 85 | 1 | 4 | 95.50 | 45 | 21 | 44 | 50.56 |
| 21–50 | 37 | 3 | 3 | 92.50 | 5 | 7 | 35 | 12.50 |
