# Supplementary material for: Benchmarking workflows to assess performance and suitability of germline variant calling pipelines in clinical diagnostic assays
Source: BMC Bioinformatics. 2021 Feb 24;22:85. doi: 10.1186/s12859-020-03934-3 (PMC7903625; doi:10.1186/s12859-020-03934-3)
Supplement: Supplementary file 6 — Additional file 6: Table S6. Benchmarking metrics for InDels of different size ranges in NA12878 (truth set NIST v3.3, total bases = 13728555) for the regions within ~7000 clinically relevant genes (as specified in Methods). [file 12859_2020_3934_MOESM6_ESM.docx]

Additional file 6: Table S6. Benchmarking metrics for InDels of different size ranges in NA12878 (truth set NIST v3.3, total bases = 13728555) for the regions within ~7000 clinically relevant genes (as specified in Methods).

| **Size of InDels in NA12878** | **Truth total** | **TP** | **FP** | **FN** | **TN** | **NPA** | **Precision** | **Recall** |
| --- | --- | --- | --- | --- | --- | --- | --- | --- |
| 1–10 | 145 | 139 | 10 | 6 | 13728400 | 100 | 93.29 | 95.86 |
| 11–20 | 7 | 7 | 0 | 0 | 13728548 | 100 | 100 | 100 |
| 21–50 | 5 | 5 | 0 | 0 | 13728550 | 100 | 100 | 100 |
| All Indels | 156 | 150 | 10 | 6 | 13728389 | 100 | 93.75 | 96.15 |
