# Supplementary material for: Benchmarking workflows to assess performance and suitability of germline variant calling pipelines in clinical diagnostic assays
Source: BMC Bioinformatics. 2021 Feb 24;22:85. doi: 10.1186/s12859-020-03934-3 (PMC7903625; doi:10.1186/s12859-020-03934-3)
Supplement: Supplementary file 7 — Additional file 7: Table S7. Benchmarking metrics for InDels of different size ranges in NA24143 (truth set NIST v3.3, total bases = 12549224) for the regions within ~7000 clinically relevant genes (as specified in Methods). [file 12859_2020_3934_MOESM7_ESM.docx]

Additional file 7: Table S7. Benchmarking metrics for InDels of different size ranges in NA24143 (truth set NIST v3.3, total bases = 12549224) for the regions within ~7000 clinically relevant genes (as specified in Methods).

| **Size of InDels in NA24143** | **Truth total** | **TP** | **FP** | **FN** | **TN** | **NPA** | **Precision** | **Recall** |
| --- | --- | --- | --- | --- | --- | --- | --- | --- |
| 1–10 | 153 | 143 | 16 | 10 | 12549055 | 100 | 89.94 | 93.46 |
| 11–20 | 8 | 8 | 0 | 0 | 12549216 | 100 | 100 | 100 |
| 21–50 | 3 | 3 | 0 | 0 | 12549221 | 100 | 100 | 100 |
| All Indels | 163 | 153 | ]16 | 10 | 12549045 | 100 | 90.53 | 93.87 |
