# Supplementary material for: Benchmarking workflows to assess performance and suitability of germline variant calling pipelines in clinical diagnostic assays
Source: BMC Bioinformatics. 2021 Feb 24;22:85. doi: 10.1186/s12859-020-03934-3 (PMC7903625; doi:10.1186/s12859-020-03934-3)
Supplement: Supplementary file 8 — Additional file 8: Table S8. Benchmarking metrics for InDels of different size ranges in NA24149 (truth set NIST v3.3, total bases = 12538042) for the regions within ~7000 clinically relevant genes (as specified in Methods). [file 12859_2020_3934_MOESM8_ESM.docx]

Additional file 8: Table S8. Benchmarking metrics for InDels of different size ranges in NA24149 (truth set NIST v3.3, total bases = 12538042) for the regions within ~7000 clinically relevant genes (as specified in Methods).

| **Size of InDels in NA24149** | **Truth total** | **TP** | **FP** | **FN** | **TN** | **NPA** | **Precision** | **Recall** |
| --- | --- | --- | --- | --- | --- | --- | --- | --- |
| 1–10 | 156 | 153 | 8 | 3 | 12537878 | 100 | 95.03 | 98.08 |
| 11–20 | 8 | 8 | 1 | 0 | 12538033 | 100 | 88.89 | 100 |
| 21–50 | 1 | 1 | 0 | 0 | 12538041 | 100 | 100 | 100 |
| All Indels | 163 | 161 | 9 | 3 | 12537869 | 100 | 94.71 | 98.16 |
