# Supplementary material for: Benchmarking workflows to assess performance and suitability of germline variant calling pipelines in clinical diagnostic assays
Source: BMC Bioinformatics. 2021 Feb 24;22:85. doi: 10.1186/s12859-020-03934-3 (PMC7903625; doi:10.1186/s12859-020-03934-3)
Supplement: Supplementary file 9 — Additional file 9: Table S9. Benchmarking metrics for InDels of different size ranges in NA24631 (truth set NIST v3.3, total bases = 12808688) for the regions within ~7000 clinically relevant genes (as specified in Methods). [file 12859_2020_3934_MOESM9_ESM.docx]

Additional file 9: Table S9. Benchmarking metrics for InDels of different size ranges in NA24631 (truth set NIST v3.3, total bases = 12808688) for the regions within ~7000 clinically relevant genes (as specified in Methods).

| **Size of InDels in NA24631** | **Truth total** | **TP** | **FP** | **FN** | **TN** | **NPA** | **Precision** | **Recall** |
| --- | --- | --- | --- | --- | --- | --- | --- | --- |
| 1–10 | 153 | 146 | 16 | 7 | 12808519 | 100 | 90.12 | 95.42 |
| 11–20 | 5 | 5 | 0 | 0 | 12808683 | 100 | 100 | 100 |
| 21–50 | 5 | 4 | 0 | 1 | 12808683 | 100 | 100 | 80 |
| All Indels | 162 | 154 | 16 | 8 | 12808510 | 100 | 90.59 | 95.06 |
