# Supplementary material for: Benchmarking workflows to assess performance and suitability of germline variant calling pipelines in clinical diagnostic assays
Source: BMC Bioinformatics. 2021 Feb 24;22:85. doi: 10.1186/s12859-020-03934-3 (PMC7903625; doi:10.1186/s12859-020-03934-3)
Supplement: Supplementary file 10 — Additional file 10: Table S10. Benchmarking metrics on the number of InDels of different size ranges in NA12878 (truth set NIST v3.3, total bases = 71152019) for the whole exome regions including non–coding exons, splice sites (+/- 20 bp) and clinically relevant deep intronic regions. [file 12859_2020_3934_MOESM10_ESM.docx]

Additional file 10: Table S10. Benchmarking metrics on the number of InDels of different size ranges in NA12878 (truth set NIST v3.3, total bases = 71152019) for the whole exome regions including non–coding exons, splice sites (+/- 20 bp) and clinically relevant deep intronic regions.

| **Size of InDels in NA12878** | **Truth total** | **TP** | **FP** | **FN** | **TN** | **NPA** | **Precision** | **Recall** |
| --- | --- | --- | --- | --- | --- | --- | --- | --- |
| 1–10 | 5108 | 4704 | 781 | 404 | 71146130 | 100 | 85.76 | 92.09 |
| 11–20 | 209 | 194 | 13 | 15 | 71151797 | 100 | 93.72 | 92.82 |
| 21–50 | 52 | 47 | 5 | 5 | 71151962 | 100 | 90.38 | 90.38 |
| All Indels | 5318 | 4910 | 800 | 424 | 71145885 | 100 | 85.99 | 92.03 |
