# Supplementary material for: Benchmarking workflows to assess performance and suitability of germline variant calling pipelines in clinical diagnostic assays
Source: BMC Bioinformatics. 2021 Feb 24;22:85. doi: 10.1186/s12859-020-03934-3 (PMC7903625; doi:10.1186/s12859-020-03934-3)
Supplement: Supplementary file 11 — Additional file 11: Table S11. Benchmarking metrics on the number of InDels of different size ranges in NA24143 (truth set NIST v3.3, total bases = 65657646) for the whole exome regions including non-coding exons, splice sites (+/- 20 bp) and clinically relevant deep intronic regions. [file 12859_2020_3934_MOESM11_ESM.docx]

Additional file 11: Table S11. Benchmarking metrics on the number of InDels of different size ranges in NA24143 (truth set NIST v3.3, total bases = 65657646) for the whole exome regions including non-coding exons, splice sites (+/- 20 bp) and clinically relevant deep intronic regions.

| **Size of InDels in NA24143** | **Truth total** | **TP** | **FP** | **FN** | **TN** | **NPA** | **Precision** | **Recall** |
| --- | --- | --- | --- | --- | --- | --- | --- | --- |
| 1–10 | 5168 | 4676 | 681 | 492 | 65651797 | 100 | 87.29 | 90.48 |
| 11–20 | 206 | 184 | 13 | 22 | 65657427 | 100 | 93.40 | 89.32 |
| 21–50 | 84 | 72 | 5 | 12 | 65657557 | 100 | 93.51 | 85.71 |
| All Indels | 5388 | 4878 | 700 | 526 | 65651542 | 100 | 87.45 | 90.24 |
