# Supplementary material for: Benchmarking workflows to assess performance and suitability of germline variant calling pipelines in clinical diagnostic assays
Source: BMC Bioinformatics. 2021 Feb 24;22:85. doi: 10.1186/s12859-020-03934-3 (PMC7903625; doi:10.1186/s12859-020-03934-3)
Supplement: Supplementary file 12 — Additional file 12: Table S12. Benchmarking metrics on the number of InDels of different size ranges in NA24149 (truth set NIST v3.3, total bases = 65597266) for the whole exome regions including non-coding exons, splice sites (+/- 20 bp) and clinically relevant deep intronic regions. [file 12859_2020_3934_MOESM12_ESM.docx]

Additional file 12: Table S12. Benchmarking metrics on the number of InDels of different size ranges in NA24149 (truth set NIST v3.3, total bases = 65597266) for the whole exome regions including non-coding exons, splice sites (+/- 20 bp) and clinically relevant deep intronic regions.

| **Size of InDels in NA24149** | **Truth total** | **TP** | **FP** | **FN** | **TN** | **NPA** | **Precision** | **Recall** |
| --- | --- | --- | --- | --- | --- | --- | --- | --- |
| 1–10 | 5096 | 4578 | 628 | 518 | 65591542 | 100 | 87.94 | 89.84 |
| 11–20 | 188 | 167 | 17 | 21 | 65597061 | 100 | 90.76 | 88.83 |
| 21–50 | 68 | 62 | 5 | 6 | 65597193 | 100 | 92.54 | 91.18 |
| All Indels | 5290 | 4763 | 651 | 545 | 65591307 | 100 | 87.98 | 89.70 |
