# Supplementary material for: Benchmarking workflows to assess performance and suitability of germline variant calling pipelines in clinical diagnostic assays
Source: BMC Bioinformatics. 2021 Feb 24;22:85. doi: 10.1186/s12859-020-03934-3 (PMC7903625; doi:10.1186/s12859-020-03934-3)
Supplement: Supplementary file 13 — Additional file 13: Table S13. Benchmarking metrics on the number of InDels of different size ranges in NA24631 (truth set NIST v3.3, total bases = 65657646) for the whole exome regions including non-coding exons, splice sites (+/- 20 bp) and clinically relevant deep intronic regions. [file 12859_2020_3934_MOESM13_ESM.docx]

Additional file 13: Table S13. Benchmarking metrics on the number of InDels of different size ranges in NA24631 (truth set NIST v3.3, total bases = 65657646) for the whole exome regions including non-coding exons, splice sites (+/- 20 bp) and clinically relevant deep intronic regions.

| **Size of InDels in NA24631** | **Truth total** | **TP** | **FP** | **FN** | **TN** | **NPA** | **Precision** | **Recall** |
| --- | --- | --- | --- | --- | --- | --- | --- | --- |
| 1–10 | 5555 | 5089 | 656 | 466 | 66982776 | 100 | 88.58 | 91.61 |
| 11–20 | 187 | 178 | 6 | 9 | 66988794 | 100 | 96.74 | 95.19 |
| 21–50 | 82 | 68 | 8 | 14 | 66988897 | 100 | 89.47 | 82.93 |
| All Indels | 5805 | 5316 | 671 | 489 | 66982511 | 100 | 88.79 | 91.58 |
