# Supplementary material for: Benchmarking workflows to assess performance and suitability of germline variant calling pipelines in clinical diagnostic assays
Source: BMC Bioinformatics. 2021 Feb 24;22:85. doi: 10.1186/s12859-020-03934-3 (PMC7903625; doi:10.1186/s12859-020-03934-3)
Supplement: Supplementary file 14 — Additional file 14: Table S14. Benchmarking metrics for NA24143 (SNPs and InDels, truth set NIST v3.3) within coding exons of ~7000 clinically relevant genes (as specified in Methods) using RTG vcfeval. [file 12859_2020_3934_MOESM14_ESM.docx]

Additional file 14: Table S14. Benchmarking metrics for NA24143 (SNPs and InDels, truth set NIST v3.3) within coding exons of ~7000 clinically relevant genes (as specified in Methods) using RTG vcfeval.

| **Threshold** | **TP-baseline** | **TP-call** | **FP** | **FN** | **Precision** | **Sensitivity** | **F-measure** |
| --- | --- | --- | --- | --- | --- | --- | --- |
| 12 | 7587 | 7587 | 27 | 21 | 99.65 | 99.72 | 99.68 |
| None | 7587 | 7587 | 27 | 21 | 99.65 | 99.72 | 99.68 |
