# Supplementary material for: Benchmarking workflows to assess performance and suitability of germline variant calling pipelines in clinical diagnostic assays
Source: BMC Bioinformatics. 2021 Feb 24;22:85. doi: 10.1186/s12859-020-03934-3 (PMC7903625; doi:10.1186/s12859-020-03934-3)
Supplement: Supplementary file 15 — Additional file 15: Table S15. Benchmarking metrics for NA24143 (SNPs and InDels, truth set NIST v3.3) in whole exome regions, including non-coding exons, splice sites (+/- 20 bp) and clinically relevant deep intronic regions using RTG vcfeval. [file 12859_2020_3934_MOESM15_ESM.docx]

Additional file 15: Table S15. Benchmarking metrics for NA24143 (SNPs and InDels, truth set NIST v3.3) in whole exome regions, including non-coding exons, splice sites (+/- 20 bp) and clinically relevant deep intronic regions using RTG vcfeval.

| **Threshold** | **TP-baseline** | **TP-call** | **FP** | **FN** | **Precision** | **Sensitivity** | **F-measure** |
| --- | --- | --- | --- | --- | --- | --- | --- |
| 2 | 60361 | 60363 | 1001 | 1170 | 98.37 | 98.10 | 98.23 |
| None | 60363 | 60365 | 1014 | 1168 | 98.35 | 98.10 | 98.22 |
