# Supplementary material for: Benchmarking workflows to assess performance and suitability of germline variant calling pipelines in clinical diagnostic assays
Source: BMC Bioinformatics. 2021 Feb 24;22:85. doi: 10.1186/s12859-020-03934-3 (PMC7903625; doi:10.1186/s12859-020-03934-3)
Supplement: Supplementary file 17 — Additional file 17: Fig S1. InDel size distribution histograms for NA24385 as generated by the benchmarking workflow for the coding exons of ~7000 clinically relevant genes and whole exome regions (as specified in Methods). [file 12859_2020_3934_MOESM17_ESM.pdf]

Additional file 17: Fig S1. InDel size distribution histograms for NA24385 as generated by the benchmarking workflow for the coding exons of ~7000 clinically relevant genes and whole exome regions (as specified in Methods).

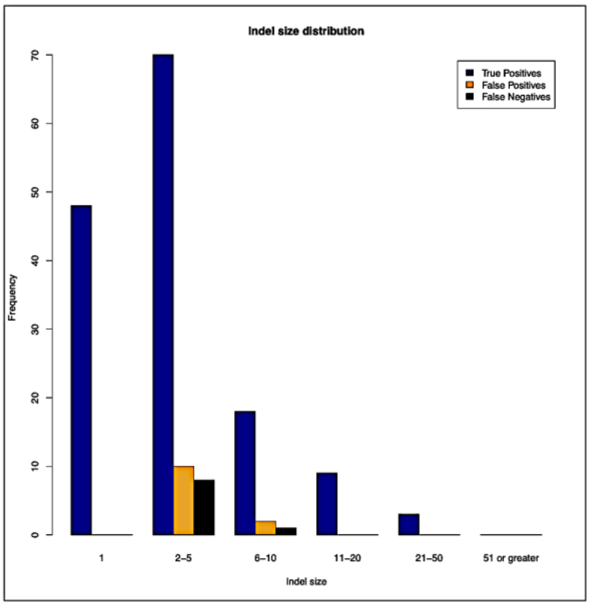

(A) NA24385 Clinically relevant regions

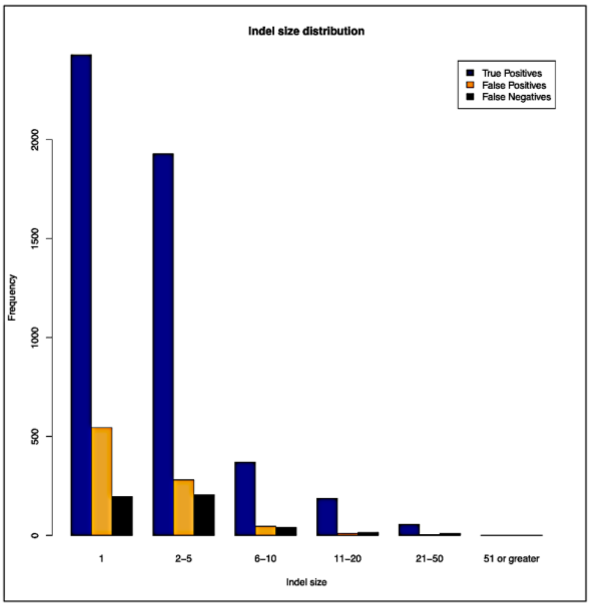

(B) NA24385 Whole exome regions
