# Supplementary material for: Benchmarking workflows to assess performance and suitability of germline variant calling pipelines in clinical diagnostic assays
Source: BMC Bioinformatics. 2021 Feb 24;22:85. doi: 10.1186/s12859-020-03934-3 (PMC7903625; doi:10.1186/s12859-020-03934-3)

Additional file 20: Fig S4. ROC curves for NA24143 in the coding exon regions (as specified in Methods) using RTG rocplot with metrics obtained from RTG vcfeval.

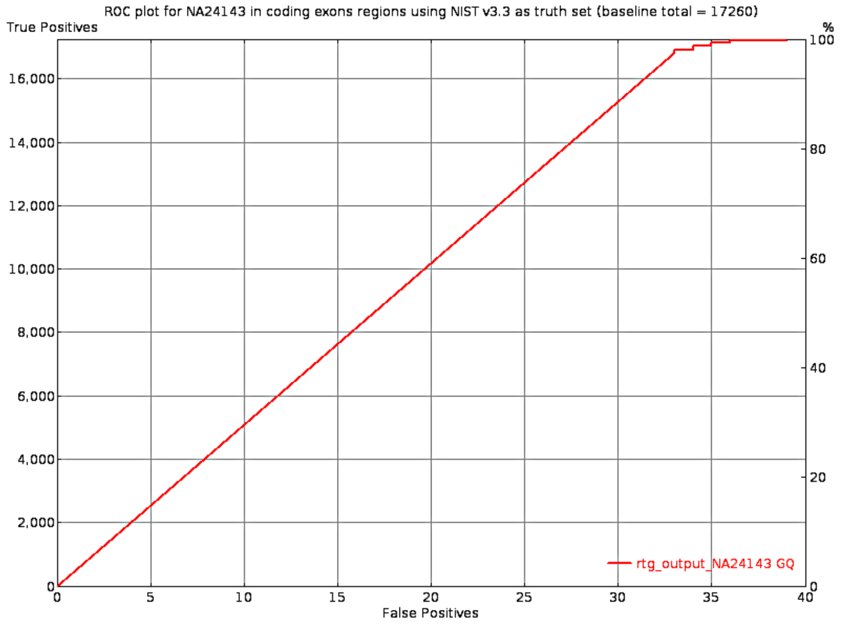

Supplement: Supplementary file 20 — Additional file 20: Fig S4. ROC curves for NA24143 in the coding exon regions (as specified in Methods) using RTG rocplot with metrics obtained from RTG vcfeval. [file 12859_2020_3934_MOESM20_ESM.pdf]
