# Supplementary material for: Benchmarking workflows to assess performance and suitability of germline variant calling pipelines in clinical diagnostic assays
Source: BMC Bioinformatics. 2021 Feb 24;22:85. doi: 10.1186/s12859-020-03934-3 (PMC7903625; doi:10.1186/s12859-020-03934-3)
Supplement: Supplementary file 25 — Additional file 25. Supplementary information. [file 12859_2020_3934_MOESM25_ESM.docx]

Benchmarking workflows to assess performance and suitability of germline variant calling pipelines in clinical diagnostic assays.

Vandhana Krishnan^1,2^, ^†^Sowmithri Utiramerur^2,3,7^, Zena Ng^3^, Somalee Datta^2,6^, Michael P. Snyder^1,2^, ^†^Euan A. Ashley^1,4,5^

Supplementary Information

***Preparation of truth sets for exome regions***

The NIST version and the FTP site used to download the original data for each of the GIAB samples (before preprocessing) used in this study are listed here.

NA12878

NIST v3.3:

ftp://ftp-trace.ncbi.nlm.nih.gov/giab/ftp/release/NA12878_HG001/NISTv3.3/NA12878_GIAB_highconf_CG-IllFB-IllGATKHC-Ion-Solid-10X_CHROM1-X_v3.3_highconf.bed

ftp://ftp-trace.ncbi.nlm.nih.gov/giab/ftp/release/NA12878_HG001/NISTv3.3/NA12878_GIAB_highconf_CG-IllFB-IllGATKHC-Ion-Solid-10X_CHROM1-X_v3.3_highconf.vcf.gz

NA24143

NIST v3.3:

ftp://ftp-trace.ncbi.nlm.nih.gov/giab/ftp/release/AshkenazimTrio/HG004_NA24143_mother/NISTv3.3/HG004_GIAB_highconf_CG-IllFB-IllGATKHC-Ion-10X_CHROM1-22_v3.3_highconf.bed

ftp://ftp-trace.ncbi.nlm.nih.gov/giab/ftp/release/AshkenazimTrio/HG004_NA24143_mother/NISTv3.3/HG004_GIAB_highconf_CG-IllFB-IllGATKHC-Ion-10X_CHROM1-22_v3.3_highconf.vcf.gz

NA24149

NIST v3.3:

ftp://ftp-trace.ncbi.nlm.nih.gov/giab/ftp/release/AshkenazimTrio/HG003_NA24149_father/NISTv3.3/HG003_GIAB_highconf_CG-IllFB-IllGATKHC-Ion-10X_CHROM1-22_v3.3_highconf.bed

ftp://ftp-trace.ncbi.nlm.nih.gov/giab/ftp/release/AshkenazimTrio/HG003_NA24149_father/NISTv3.3/HG003_GIAB_highconf_CG-IllFB-IllGATKHC-Ion-10X_CHROM1-22_v3.3_highconf.vcf.gz

NA24385

NIST v3.3:

ftp://ftp-trace.ncbi.nlm.nih.gov/giab/ftp/release/AshkenazimTrio/HG002_NA24385_son/NISTv3.3/HG002_GIAB_highconf_CG-IllFB-IllGATKHC-Ion-Solid-10X_CHROM1-22_v3.3_highconf.bed

ftp://ftp-trace.ncbi.nlm.nih.gov/giab/ftp/release/AshkenazimTrio/HG002_NA24385_son/NISTv3.3/HG002_GIAB_highconf_CG-IllFB-IllGATKHC-Ion-Solid-10X_CHROM1-22_v3.3_highconf.vcf.gz

NA24631

NIST v3.3.2:

ftp://ftp-trace.ncbi.nlm.nih.gov/giab/ftp/release/ChineseTrio/HG005_NA24631_son/NISTv3.3.2/GRCh37/HG005_GRCh37_highconf_CG-IllFB-IllGATKHC-Ion-SOLID_CHROM1-22_v.3.3.2_highconf_noMetaSV.bed

ftp://ftp-trace.ncbi.nlm.nih.gov/giab/ftp/release/ChineseTrio/HG005_NA24631_son/NISTv3.3.2/GRCh37/HG005_GRCh37_highconf_CG-IllFB-IllGATKHC-Ion-SOLID_CHROM1-22_v.3.3.2_highconf.vcf.gz

***Bash command to compute total number of bases in a region of interest***

awk '{a=$3-$2;print a}' <Consolidated.bed> | paste -sd+ - | bc

In the above command, *<Consolidated.bed>* refers to GIAB original high confidence bed file for a sample intersected with the bed file of the region of interest such as coding exons, whole exome or clinically relevant gene regions. The user can use this command to calculate bases with their desired stratified region in the bed format which is required to compute metrics such as true negatives.

***Output files generated by Benchmarking workflow***

Our benchmarking workflow generates the following output files:

1. <*Output file common prefix*>_<*Sample ID*>_CodingExons.vcf.gz

2. <*Output file common prefix*>_< *Sample ID*>_CodingExons.vcf.gz.tbi

3. <*Output file common prefix*>_< *Sample ID*>_CodingExons_counts.csv

4. <*Output file common prefix*>_<*Sample ID*>_CodingExons_counts.json

5. <*Output file common prefix*>_<*Sample ID*>_CodingExons_summary.csv

6. <*Output file common prefix*>_<*Sample ID*>_CodingExons_extended.csv

7. <*Output file common prefix*>_<*Sample ID*>_CodingExons_metrics.json

8. <*Output file common prefix*>_<*Sample ID*>_CodingExons_ConsoleOutput.txt

9. <*Output file common prefix*>_<*Sample ID*>_CodingExons_indelSizeDistribution.txt

10. <*Output file common prefix*>_<*Sample ID*>_CodingExons_indelSizeDistributionOnPlot.pdf

There is a final performance assay report generated in the form of a tab delimited file as below:

Final_benchmarking_metrics_*<current_date>.*txt

Another set of 10 files as seen above corresponding to the whole exome regions are generated.

The benchmarking framework generates the following intermediate files:

1. <<*Output file common prefix*>_<*Sample ID*>_CodingExons_SNPs_TPonly.vcf.gz

2. <*Output file common prefix*>_<*Sample ID*>_CodingExons_SNPs_FPonly.vcf.gz

3. <*Output file common prefix*>_<*Sample ID*>_CodingExons_SNPs_FNonly.vcf.gz

4. <*Output file common prefix*>_<*Sample ID*>_CodingExons_INDELs_TPonly.vcf.gz

5. <*Output file common prefix*>_<*Sample ID*>_CodingExons_INDELs_FPonly.vcf.gz

6. <*Output file common prefix*>_<*Sample ID*>_CodingExons_INDELs_FNonly.vcf.gz

7. <*Output file common prefix*>_<*Sample ID*>_CodingExons_indelDistribution.txt

Another set of 7 files as seen above corresponding to the whole exome regions are generated.

**Additional files:** .

Additional file 1: Table S1. Benchmarking metrics on SNPs and multiple nucleotide polymorphisms (MNPs) in NA24149 (truth set NIST v3.3) for the RefSeq coding exons regions generated for both the GATK and SpeedSeq pipelines that were executed using workflows run by Loom (in-house workflow engine).

| **Workflow run using Loom** | **TP** | **FP** | **FN** | **Precision** | **Recall** |
| --- | --- | --- | --- | --- | --- |
| GATK HaploypeCaller (Broad’s best practices pipeline) | 17501 | 111 | 103 | 99.37 | 99.41 |
| SpeedSeq | 17496 | 102 | 121 | 99.42 | 99.31 |

Additional file 2: Table S2. Benchmarking metrics on InDels in NA24149 (truth set NIST v3.3) for the RefSeq coding exons regions generated for both the GATK and SpeedSeq pipelines that were executed using workflows run by Loom (in-house workflow engine).

| **Workflow run using Loom** | **TP** | **FP** | **FN** | **Precision** | **Recall** |
| --- | --- | --- | --- | --- | --- |
| GATK HaploypeCaller (Broad’s best practices pipeline) | 402 | 27 | 13 | 93.70 | 96.84 |
| SpeedSeq | 367 | 17 | 45 | 95.57 | 89.07 |

Additional file 3: Table S3. Benchmarking metrics on SNPs and multiple nucleotide polymorphisms (MNPs) in NA24143 (truth set NIST v3.3) for the RefSeq coding exons regions generated for both the GATK and SpeedSeq pipelines that were executed using workflows run by Loom (in-house workflow engine).

| **Workflow run using Loom** | **TP** | **FP** | **FN** | **Precision** | **Recall** |
| --- | --- | --- | --- | --- | --- |
| GATK HaploypeCaller (Broad’s best practices pipeline) | 17542 | 91 | 98 | 99.48 | 99.44 |
| SpeedSeq | 17527 | 54 | 119 | 99.69 | 99.32 |

Additional file 4: Table S4. Benchmarking metrics on InDels in NA24143 (truth set NIST v3.3) for the RefSeq coding exons regions generated for both the GATK and SpeedSeq pipelines that were executed using workflows run by Loom (in-house workflow engine).

| **Workflow run using Loom** | **TP** | **FP** | **FN** | **Precision** | **Recall** |
| --- | --- | --- | --- | --- | --- |
| GATK HaploypeCaller (Broad’s best practices pipeline) | 424 | 21 | 19 | 95.28 | 95.67 |
| SpeedSeq | 379 | 17 | 60 | 95.70 | 86.33 |

Additional file 5: Table S5. Benchmarking metrics on InDel size distribution in NA24631 (truth set NIST v3.3.2) for whole exome regions, including non-coding exons, splice sites (+/- 2 bp) and clinically relevant deep intronic regions intersected with clinical exome to assess performance of GATK and SpeedSeq pipelines.

| **Indel size** | **GATK HaploypeCaller**  **(Broad’s best practices pipeline)** | | | | **SpeedSeq** | | | |
| --- | --- | --- | --- | --- | --- | --- | --- | --- |
|  | TP | FP | FN | Recall | TP | FP | FN | Recall |
| 1 | 1049 | 120 | 78 | 93.07 | 1065 | 32 | 68 | 93.99 |
| 2–5 | 858 | 112 | 78 | 91.66 | 803 | 51 | 133 | 85.79 |
| 6–10 | 194 | 10 | 7 | 96.51 | 158 | 17 | 48 | 76.69 |
| 11–20 | 85 | 1 | 4 | 95.50 | 45 | 21 | 44 | 50.56 |
| 21–50 | 37 | 3 | 3 | 92.50 | 5 | 7 | 35 | 12.50 |

Additional file 6: Table S6. Benchmarking metrics for InDels of different size ranges in NA12878 (truth set NIST v3.3, total bases = 13728555) for the regions within ~7000 clinically relevant genes (as specified in Methods).

| **Size of InDels in NA12878** | **Truth total** | **TP** | **FP** | **FN** | **TN** | **NPA** | **Precision** | **Recall** |
| --- | --- | --- | --- | --- | --- | --- | --- | --- |
| 1–10 | 145 | 139 | 10 | 6 | 13728400 | 100 | 93.29 | 95.86 |
| 11–20 | 7 | 7 | 0 | 0 | 13728548 | 100 | 100 | 100 |
| 21–50 | 5 | 5 | 0 | 0 | 13728550 | 100 | 100 | 100 |
| All Indels | 156 | 150 | 10 | 6 | 13728389 | 100 | 93.75 | 96.15 |

Additional file 7: Table S7. Benchmarking metrics for InDels of different size ranges in NA24143 (truth set NIST v3.3, total bases = 12549224) for the regions within ~7000 clinically relevant genes (as specified in Methods).

| **Size of InDels in NA24143** | **Truth total** | **TP** | **FP** | **FN** | **TN** | **NPA** | **Precision** | **Recall** |
| --- | --- | --- | --- | --- | --- | --- | --- | --- |
| 1–10 | 153 | 143 | 16 | 10 | 12549055 | 100 | 89.94 | 93.46 |
| 11–20 | 8 | 8 | 0 | 0 | 12549216 | 100 | 100 | 100 |
| 21–50 | 3 | 3 | 0 | 0 | 12549221 | 100 | 100 | 100 |
| All Indels | 163 | 153 | ]16 | 10 | 12549045 | 100 | 90.53 | 93.87 |

Additional file 8: Table S8. Benchmarking metrics for InDels of different size ranges in NA24149 (truth set NIST v3.3, total bases = 12538042) for the regions within ~7000 clinically relevant genes (as specified in Methods).

| **Size of InDels in NA24149** | **Truth total** | **TP** | **FP** | **FN** | **TN** | **NPA** | **Precision** | **Recall** |
| --- | --- | --- | --- | --- | --- | --- | --- | --- |
| 1–10 | 156 | 153 | 8 | 3 | 12537878 | 100 | 95.03 | 98.08 |
| 11–20 | 8 | 8 | 1 | 0 | 12538033 | 100 | 88.89 | 100 |
| 21–50 | 1 | 1 | 0 | 0 | 12538041 | 100 | 100 | 100 |
| All Indels | 163 | 161 | 9 | 3 | 12537869 | 100 | 94.71 | 98.16 |

Additional file 9: Table S9. Benchmarking metrics for InDels of different size ranges in NA24631 (truth set NIST v3.3, total bases = 12808688) for the regions within ~7000 clinically relevant genes (as specified in Methods).

| **Size of InDels in NA24631** | **Truth total** | **TP** | **FP** | **FN** | **TN** | **NPA** | **Precision** | **Recall** |
| --- | --- | --- | --- | --- | --- | --- | --- | --- |
| 1–10 | 153 | 146 | 16 | 7 | 12808519 | 100 | 90.12 | 95.42 |
| 11–20 | 5 | 5 | 0 | 0 | 12808683 | 100 | 100 | 100 |
| 21–50 | 5 | 4 | 0 | 1 | 12808683 | 100 | 100 | 80 |
| All Indels | 162 | 154 | 16 | 8 | 12808510 | 100 | 90.59 | 95.06 |

Additional file 10: Table S10. Benchmarking metrics on the number of InDels of different size ranges in NA12878 (truth set NIST v3.3, total bases = 71152019) for the whole exome regions including non–coding exons, splice sites (+/- 20 bp) and clinically relevant deep intronic regions.

| **Size of InDels in NA12878** | **Truth total** | **TP** | **FP** | **FN** | **TN** | **NPA** | **Precision** | **Recall** |
| --- | --- | --- | --- | --- | --- | --- | --- | --- |
| 1–10 | 5108 | 4704 | 781 | 404 | 71146130 | 100 | 85.76 | 92.09 |
| 11–20 | 209 | 194 | 13 | 15 | 71151797 | 100 | 93.72 | 92.82 |
| 21–50 | 52 | 47 | 5 | 5 | 71151962 | 100 | 90.38 | 90.38 |
| All Indels | 5318 | 4910 | 800 | 424 | 71145885 | 100 | 85.99 | 92.03 |

Additional file 11: Table S11. Benchmarking metrics on the number of InDels of different size ranges in NA24143 (truth set NIST v3.3, total bases = 65657646) for the whole exome regions including non-coding exons, splice sites (+/- 20 bp) and clinically relevant deep intronic regions.

| **Size of InDels in NA24143** | **Truth total** | **TP** | **FP** | **FN** | **TN** | **NPA** | **Precision** | **Recall** |
| --- | --- | --- | --- | --- | --- | --- | --- | --- |
| 1–10 | 5168 | 4676 | 681 | 492 | 65651797 | 100 | 87.29 | 90.48 |
| 11–20 | 206 | 184 | 13 | 22 | 65657427 | 100 | 93.40 | 89.32 |
| 21–50 | 84 | 72 | 5 | 12 | 65657557 | 100 | 93.51 | 85.71 |
| All Indels | 5388 | 4878 | 700 | 526 | 65651542 | 100 | 87.45 | 90.24 |

Additional file 12: Table S12. Benchmarking metrics on the number of InDels of different size ranges in NA24149 (truth set NIST v3.3, total bases = 65597266) for the whole exome regions including non-coding exons, splice sites (+/- 20 bp) and clinically relevant deep intronic regions.

| **Size of InDels in NA24149** | **Truth total** | **TP** | **FP** | **FN** | **TN** | **NPA** | **Precision** | **Recall** |
| --- | --- | --- | --- | --- | --- | --- | --- | --- |
| 1–10 | 5096 | 4578 | 628 | 518 | 65591542 | 100 | 87.94 | 89.84 |
| 11–20 | 188 | 167 | 17 | 21 | 65597061 | 100 | 90.76 | 88.83 |
| 21–50 | 68 | 62 | 5 | 6 | 65597193 | 100 | 92.54 | 91.18 |
| All Indels | 5290 | 4763 | 651 | 545 | 65591307 | 100 | 87.98 | 89.70 |

Additional file 13: Table S13. Benchmarking metrics on the number of InDels of different size ranges in NA24631 (truth set NIST v3.3, total bases = 65657646) for the whole exome regions including non-coding exons, splice sites (+/- 20 bp) and clinically relevant deep intronic regions.

| **Size of InDels in NA24631** | **Truth total** | **TP** | **FP** | **FN** | **TN** | **NPA** | **Precision** | **Recall** |
| --- | --- | --- | --- | --- | --- | --- | --- | --- |
| 1–10 | 5555 | 5089 | 656 | 466 | 66982776 | 100 | 88.58 | 91.61 |
| 11–20 | 187 | 178 | 6 | 9 | 66988794 | 100 | 96.74 | 95.19 |
| 21–50 | 82 | 68 | 8 | 14 | 66988897 | 100 | 89.47 | 82.93 |
| All Indels | 5805 | 5316 | 671 | 489 | 66982511 | 100 | 88.79 | 91.58 |

Additional file 14: Table S14. Benchmarking metrics for NA24143 (SNPs and InDels, truth set NIST v3.3) within coding exons of ~7000 clinically relevant genes (as specified in Methods) using RTG vcfeval.

| **Threshold** | **TP-baseline** | **TP-call** | **FP** | **FN** | **Precision** | **Sensitivity** | **F-measure** |
| --- | --- | --- | --- | --- | --- | --- | --- |
| 12 | 7587 | 7587 | 27 | 21 | 99.65 | 99.72 | 99.68 |
| None | 7587 | 7587 | 27 | 21 | 99.65 | 99.72 | 99.68 |

Additional file 15: Table S15. Benchmarking metrics for NA24143 (SNPs and InDels, truth set NIST v3.3) in whole exome regions, including non-coding exons, splice sites (+/- 20 bp) and clinically relevant deep intronic regions using RTG vcfeval.

| **Threshold** | **TP-baseline** | **TP-call** | **FP** | **FN** | **Precision** | **Sensitivity** | **F-measure** |
| --- | --- | --- | --- | --- | --- | --- | --- |
| 2 | 60361 | 60363 | 1001 | 1170 | 98.37 | 98.10 | 98.23 |
| None | 60363 | 60365 | 1014 | 1168 | 98.35 | 98.10 | 98.22 |

Additional file 16: Table S16. Benchmarking metrics for NA24143 (SNPs and InDels, truth set NIST v3.3) in the RefSeq coding exon regions using RTG vcfeval.

| **Threshold** | **TP-baseline** | **TP-call** | **FP** | **FN** | **Precision** | **Sensitivity** | **F-measure** |
| --- | --- | --- | --- | --- | --- | --- | --- |
| 6 | 17221 | 17220 | 38 | 39 | 99.78 | 99.77 | 99.78 |
| None | 17221 | 17220 | 39 | 39 | 99.77 | 99.77 | 99.77 |

Additional file 17: Fig S1. InDel size distribution histograms for NA24385 as generated by the benchmarking workflow for the coding exons of ~7000 clinically relevant genes and whole exome regions (as specified in Methods).


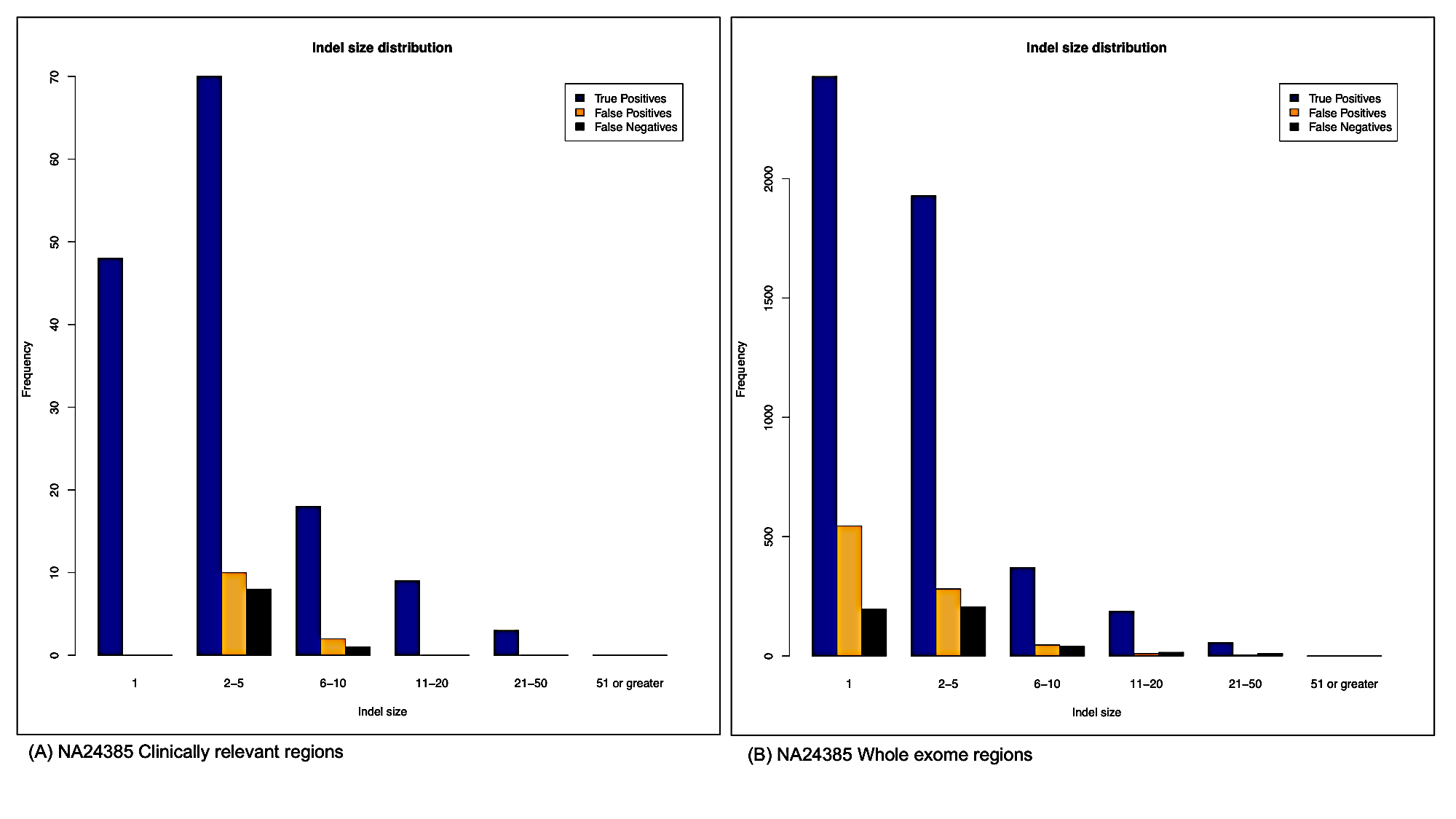


Additional file 18: Fig S2. ROC curves for NA24143 within coding exons of ~7000 clinically relevant genes using RTG rocplot with metrics obtained from RTG vcfeval.


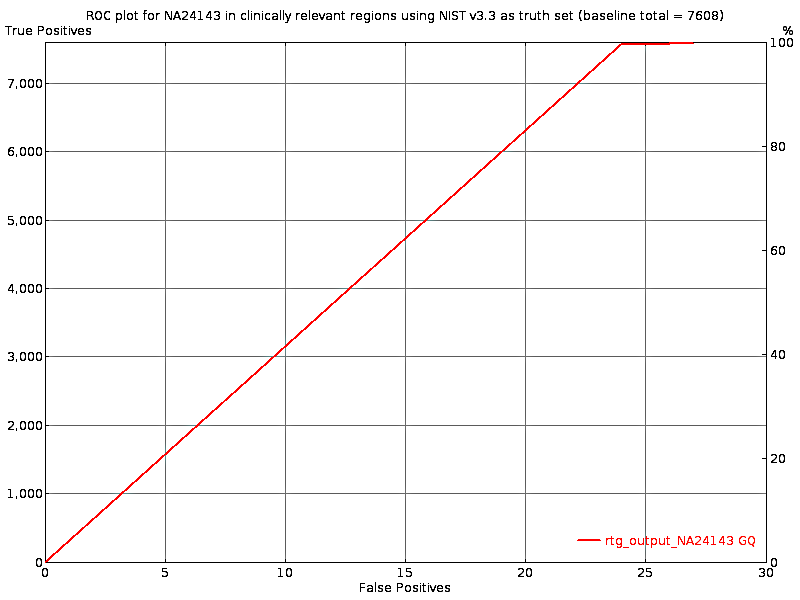


Additional file 19: Fig S3. ROC curves for NA24143 in the whole exome regions (as specified in Methods) using RTG rocplot with metrics obtained from RTG vcfeval.

**
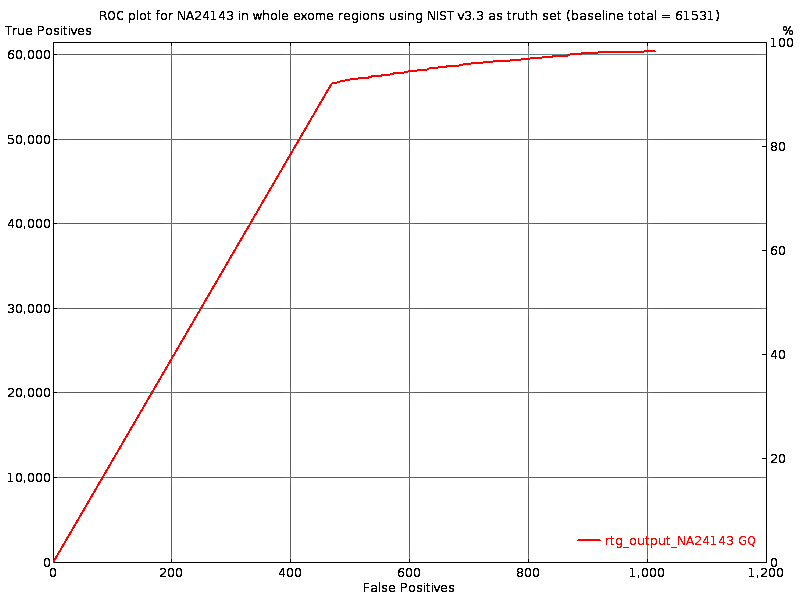
**

Additional file 20: S4. ROC curves for NA24143 in the coding exon regions (as specified in Methods) using RTG rocplot with metrics obtained from RTG vcfeval.

**
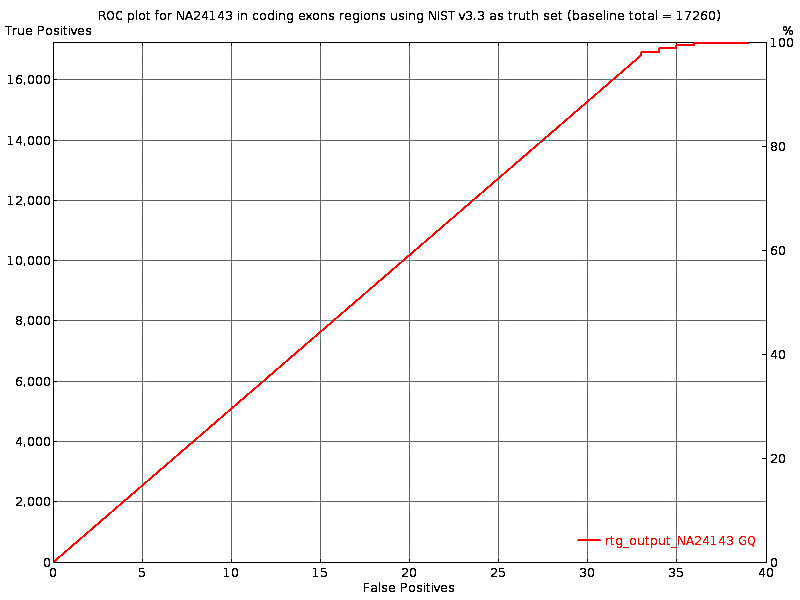
**

The following additional files 21, 22, 23 and 24 can also be found in our GitHub repository —  *https://github.com/StanfordBioinformatics/stanford-benchmarking-workflows.git*

Additional file 21: File 1. indelSizeDistribution_Detailed.R

Additional file 22: File 2. benchmarking_truth_set.py

Additional file 23: File 3. verify_variants.py

Additional file 24: File 4. Final_benchmarking_metrics.txt
